# Chronic inflammation role in the obesity-diabetes association: a case-cohort study

**DOI:** 10.1186/1758-5996-5-31

**Published:** 2013-06-27

**Authors:** Vivian C Luft, Maria I Schmidt, James S Pankow, David Couper, Christie M Ballantyne, J Hunter Young, Bruce B Duncan

**Affiliations:** 1 Graduate Studies Program in Epidemiology, School of Medicine, Federal University of Rio Grande do Sul, Porto Alegre, RS, Brazil; 2 Department of Epidemiology, Gillings School of Global Public Health, University of North Carolina, Chapel Hill, NC, United States of America; 3 Division of Epidemiology & Community Health, School of Public Health, University of Minnesota, Minneapolis, MN, United States of America; 4 Department of Biostatistics, Gillings School of Global Public Health, University of North Carolina, Chapel Hill, NC, United States of America; 5 Department of Medicine, Baylor College of Medicine, Houston, TX, United States of America; 6 Departments of Medicine and Epidemiology, The Johns Hopkins University, Baltimore, MD, United States of America

**Keywords:** Diabetes, Obesity, Inflammation, Adipokines, Humans, Epidemiologic studies

## Abstract

**Background:**

Chronic inflammation is related to both obesity and diabetes. Our aim was to investigate to what extent this inflammation contributes to the association between obesity and diabetes.

**Methods:**

Using a case-cohort design, we followed 567 middle-aged individuals who developed diabetes and 554 who did not over 9 years within the ARIC Study. Weighted Cox proportional hazards analyses permitted statistical inference to the entire cohort.

**Results:**

Obese individuals (BMI≥30 kg/m^2^), compared to those with BMI<25 kg/m^2^, presented a large increased risk of developing diabetes (HR[obesity]=6.4, 95%CI 4.5–9.2), as did those in the highest (compared to the lowest) quartile of waist circumference (HR[waist]=8.3, 95%CI 5.6–12.3), in analyses adjusted for age, gender, ethnicity, study center, and parental history of diabetes. Notably, further adjustment for adiponectin and inflammation markers halved the magnitude of these associations (HR[obesity]=3.2, 95%CI 2.1–4.7; and HR[waist]=4.2, 95%CI 2.8–6.5). In similar modeling, attenuation obtained by adding fasting insulin, instead of these markers, was only slightly more pronounced HR[obesity]=2.7, 95%CI 1.7–4.1; and HR[waist]=3.6, 95%CI 2.3–5.8).

**Conclusions:**

The marked decrease in the obesity-diabetes association after taking into account inflammation markers and adipokines indicates their major role in the pathways leading to adult onset of diabetes in obese individuals.

## Background

Diabetes is a health problem of increasing concern. The World Health Organization estimates that 2.9 million deaths per year are attributable to diabetes, and this number is likely to increase by more than 50% in the next decade [1]. Much of this burden may be explained by the dramatic rise in obesity rates [2]. It is well established that obesity is one of the strongest risk factors for diabetes, but causal mechanisms for this relationship are not fully established [3].

Obesity has been shown to lead to functional and morphological damage to remote tissues via circulating factors [3-5]. Hotasmiligil first proposed, in 1994, that inflammation is a key component of the obesity-diabetes link [6]. In the Atherosclerosis Risk in Communities (ARIC) study, we demonstrated that a mild state of inflammation precedes and predicts type 2 diabetes, independently of other risk factors, including obesity [7]. The last decade has witnessed a considerable number of investigations in humans and animals to support the role of inflammation and adipokines in the development of diabetes [8]. However, to our knowledge, to what degree inflammatory processes and insulin resistance contribute to the association of obesity and diabetes has not been evaluated in an epidemiologic context.

The ARIC study offers a unique opportunity to examine this through a case-cohort study rich in measurements of inflammation markers and adipokines. Thus, we investigated the possible role of inflammatory processes, as indicated by biomarkers, and adipokines in the obesity-diabetes association, by quantifying the degree of reduction in the strength of the obesity-diabetes association when taking these factors into account.

## Methods

### Subjects

Between 1987 and 1989 the Atherosclerosis Risk in Communities (ARIC) study recruited a population-based cohort of 15,792 men and women aged 45–64 years from four US communities. In the present analyses, we used a case-cohort design, as previously described in the investigation of the role of several inflammation biomarkers in the development of diabetes in the ARIC study [9-11]. Prior to sampling, we excluded 2,018 participants with prevalent diabetes, 95 members of minority ethnic groups with small numbers, 853 individuals who did not return to any follow-up visit, 26 with no valid diabetes determination at follow-ups, 7 with restrictions on stored plasma use, 12 with missing baseline anthropometric measurements, and 2,506 participants in previous ARIC case–control and case-cohort studies involving cardiovascular disease for whom stored plasma was either previously exhausted or held in reserve. We selected ethnicity-stratified (50% white, 50% African-American) random samples of both cases of incident diabetes and members of the full ARIC cohort not presenting diabetes at baseline, resulting in 1198 individuals. We then additionally excluded 45 participants with incomplete fasting (<8 h) and 32 not having values for inflammation markers or non-esterified fatty acids. Our final sample was 567 diabetes cases and 554 non-cases. A few of the incident cases of diabetes overlapped with and some were selected only via the cohort random sample (n = 650). Cases were defined on the basis of a reported physician diagnosis, reported use of antidiabetic medications, or a fasting glucose value ≥7.0 mmol/l, at any of the 3 follow-up visits, each occurring approximately 3 years after the previous. The date of diabetes incidence was estimated by linear interpolation using glucose values at the ascertaining visit and the prior one, as previously described [9]. Non-cases were followed for a median of 9 years, and incident cases were followed until the onset of diabetes (median = 3 years). Human-subject research review committee at the Hospital de Clínicas de Porto Alegre approved this study, and all participants gave written informed consent.

### Measurements

We measured waist girth at the umbilical level and hip circumference at the maximum hip girth in order to obtain the waist-to-hip ratio, and divided weight by height squared to obtain the body mass index (BMI). Individuals presenting BMI ≥ 30 kg/m^2^ were classified as obese, those presenting a BMI value equal or greater than 25 but lower than 30 kg/m^2^ were classified as overweight, and those with BMI < 25 kg/m^2^ (including 2 individuals < 18.5 kg/m^2^) were considered as having normal body weight. In order to assess the association of waist circumference values with incidence of diabetes, we also compared waist circumference extreme quartiles.

We defined parental history of diabetes as a report of diabetes in either parent, and hypertension as sitting blood pressure of 140/90 mm Hg or more or use of antihypertension medication.

We used a score [9], ranging from 0 to 6, to indicate low-grade systemic inflammation, attributing 1 point for a value greater than the median of the cohort sample for each of the six inflammation markers: IL-6, C-reactive protein, orosomucoid, sialic acid, white cell count and fibrinogen. Laboratory measurements and reliability coefficients for these measurements are described in previous reports [9-12]. Complement component 3 was measured by immunoturbidimetric assay, with reliability coefficient of 0.67.

### Statistical analysis

We used weighted Spearman correlations to describe crude associations between BMI and waist circumference and other variables, and weighted ANCOVA to compute adjusted BMI means in diabetes cases and non-cases. We performed Cox proportional hazards regressions to analyze the relation between BMI and time to onset of diabetes, with appropriate weighting for the stratified sample selection. In these analyses, weights are defined as the inverse of the ethnicity-specific sampling fractions. Leptin quartiles were defined in a sex-specific manner [11]. Adiponectin, C3, oxidized LDL, ICAM-1 and other continuous variables were centered in their means, in order to avoid multicollinearity. Centering was performed subtracting from each observed value the mean of the variable in question and then dividing the difference by the standard deviation of the variable’s distribution: (observed – mean) / standard deviation. Thus, values of continuous variables are expressed as standard deviations from the mean. To test heterogeneity in the obesity–incident diabetes association across categories of covariates of ethnicity (African American vs. white) and smoking (current vs. former vs. never), shown in previous analyses to modify the association between inflammation markers and diabetes [9], we employed a test of interaction. Statistical analyses were performed using the SAS (SAS Institute Inc., Cary, NC) and SUDAAN (Research Triangle Institute, Raleigh, NC) statistical software packages, based on the case-cohort sampling design. The proportional hazards assumption was examined through plots of Martingale and Schoenfeld residuals [13]. Collinearity across independent variables was investigated with linear regression models: variance inflation factors were < 2.5 in BMI models, < 4.5 in waist models and < 3.5 in insulin models.

## Results

Characteristics of cases and non-cases have previously been reported [11]. In brief, the cohort was made up of U.S. adults aged 45–64 years. According to the BMI criteria, 31% of the individuals of the cohort random sample (n=650) were at normal body weight (18.5 < BMI < 25 kg/m^2^), 41% were overweight (25 ≤ BMI < 30 kg/m^2^) and 28% were obese (BMI ≥ 30 kg/m2) at baseline. BMI mean values were 26.9 (95%CI: 26.4–27.5) for men, 26.8 (95%CI 26.4–27.4) kg/m^2^ for women, and were higher in African-Americans (28.8, 95%CI 28.1–29.5 kg/m^2^) than whites (26.4, 95%CI 25.9–26.9 kg/m^2^). According to the criteria of the Third Report of the National Cholesterol Education Program (NCEP) criteria [14], 52% of the individuals in the cohort random sample presented abdominal obesity at baseline (waist circumference >102 cm for men and >88 cm for women). In this population, the highest quartile of waist circumference was >102 cm in men and >101 cm in women, and the first quartile of waist circumference was <91 cm in men and <84 cm in women.

Spearman correlations, assessed in the cohort random sample (Table 1), show that BMI, waist circumference and serum insulin levels were associated with virtually all of the inflammation markers and adipokines. Correlations were frequently of the magnitude seen for metabolic factors.

**Table 1:** Spearman correlations for obesity with inflammation and metabolic variables in the cohort random sample (n=650)

	**BMI**		**Waist**		**Insulin**	
	**r**	**P-value**	**r**	**P-value**	**r**	**P-value**
Main exposure variables						
BMI			0.83	<0.01	0.55	<0.01
Waist	0.83	<0.01			0.53	<0.01
Insulin	0.55	<0.01	0.53	<0.01		
Inflammation markers and adipokines						
Adiponectin, in men	−0.23	<0.01	−0.25	<0.01	−0.30	<0.01
Adiponectin, in women	−0.31	<0.01	−0.29	<0.01	−0.46	<0.01
C-reactive protein	0.40	<0.01	0.36	<0.01	0.35	<0.01
IL-6	0.29	<0.01	0.32	<0.01	0.19	<0.01
Fibrinogen	0.24	<0.01	0.22	<0.01	0.20	<0.01
Orosomucoid	0.32	<0.01	0.33	<0.01	0.31	<0.01
Sialic acid	0.19	<0.01	0.16	<0.01	0.11	0.01
White blood count	0.07	<0.01	0.14	<0.01	0.10	<0.01
Complement component 3	0.43	<0.01	0.39	<0.01	0.46	0.06
Oxidized LDL	0.06	0.12	0.10	0.01	0.07	0.06
ICAM-1 (ng/mL)	0.04	0.36	0.12	<0.01	0.10	0.01
Leptin, in men	0.64	<0.01	0.65	<0.01	0.50	<0.01
Leptin, in women	0.73	<0.01	0.69	<0.01	0.65	<0.01
Other metabolic variables						
Non-esterified fatty acids	0.07	0.07	0.04	0.31	−0.01	0.75
Systolic blood pressure	0.24	<0.01	0.24	<0.01	0.33	<0.01
Diastolic blood pressure	0.21	<0.01	0.20	<0.01	0.27	<0.01
Triglycerides	0.21	<0.01	0.28	<0.01	0.30	<0.01
HDL-cholesterol, in men	−0.18	0.01	−0.26	<0.01	−0.28	<0.01
HDL-cholesterol, in women	−0.28	<0.01	−0.32	<0.01	−0.36	<0.01
HOMA-IR	0.55	<0.01	0.53	<0.01	0.99	<0.01
Fasting glucose	0.26	<0.01	0.30	<0.01	0.44	<0.01

Table 2 shows progressive parallel models for diabetes, considering obesity and overweight (according to BMI, both categories in the same model), waist circumference and fasting insulin as main exposure variables. Obesity, compared to normal body weight, was a strong risk factor for diabetes (HR=6.4, 95%CI 4.5–9.2), adjusted for age, gender, ethnicity, study center, and parental history of diabetes. Inclusion of adiponectin and then other adipokines and inflammation markers greatly reduced the magnitude of the association (HR=4.7, 95%CI 3.2–6.8 and 3.2, 95%CI 2.1–4.7, respectively). A similar pattern of associations was seen when overweight (25 kg/m^2^ ≤ BMI < 30 kg/m^2^) and waist circumference were the main exposure variables. Although the same pattern was also observed when fasting insulin was the main exposure, the minimally adjusted hazard ratio was somewhat larger and the reduction with adiponectin adjustment, more pronounced. After including the adipokine leptin in these analyses, the association increased slightly, particularly so in the waist circumference analysis. When additionally adjusting for hypertension and other variables associated with the metabolic syndrome, associations were further reduced.

**Table 2:** Hazards ratios for diabetes according to obesity and insulin levels, considering possible mediators and confounders

	**Main exposure**			
	**Overweight **^**a**^	**Obesity **^**b**^	**Waist circumference 4**^**th**^**quartile **^**c**^	**Insulin 4**^**th**^**quartile **^**d**^
	**HR (95%CI)**	**HR (95%CI)**	**HR (95%CI)**	**HR (95%CI)**
Model 1	2.07 (1.45–2.95)	6.43 (4.48–9.21)	8.30 (5.60–12.28)	10.71 (6.20–18.51)
Model 2	1.72 (1.20–2.48)	4.69 (3.23–6.81)	6.27 (4.18–9.40)	6.90 (3.87–12.32)
Model 3	1.45 (1.00–2.11)	3.18 (2.14–4.73)	4.23 (2.76–6.49)	4.44 (2.45–8.04)
Model 4	1.46 (0.98–2.17)	3.21 (2.01–5.14)	5.08 (2.97–8.71)	4.56 (2.38–8.73)
Model 5	1.17 (0.78–1.76)	2.26 (1.37–3.73)	3.19 (1.58–6.45)	3.42 (1.75–6.71)

Model 1: Adjusted for age, study center, ethnicity, gender, and family history of diabetes.

Model 2: Model 1 + adiponectin.

Model 3: Model 2 + inflammation score, C3; oxidized LDL, ICAM-1.

Model 4: Model 3 + leptin (gender-specific quartiles).

Model 5: Model 4 + hypertension, non-esterified fatty acids, ln-triglycerides, ln-triglycerides^2^, HDL-cholesterol, BMI, BMI^2^, waist-to-hip ratio, ln-insulin (those not already in the model).

^a^ Hazards ratio of overweight (25 kg/m^2^ ≤ BMI < 30 kg/m^2^) versus BMI < 25 kg/m^2^.

^b^ Hazards ratio of obese (BMI ≥ 30 kg/m^2^) versus BMI < 25 kg/m^2^.

^c^ 4^th^ vs. 1^st^ quartile. In men: waist circumference >102 cm vs. <91 cm; in women: waist circumference >101 cm vs. <84 cm.

^d^ 4^th^ vs. 1^st^ quartile: >13 μu/mL vs. < 5 μu/mL.

As insulin resistance is considered a central mechanism in the causation of type 2 diabetes, we performed similar models of progressive adjustment, but adding insulin prior to adiponectin and inflammation markers. As seen in Table 3, insulin adjustment markedly decreased the size of the obesity-diabetes association. Nonetheless, further adjustment for adiponectin and inflammation markers led to additional large decreases – HR[obesity] from 2.7, 1.7–4.1 to 1.8, 1.2–2.9 and HR[waist circumference] from 3.6, 2.3–5.8 to 2.5, 1.6–4.1). Adjustment with the HOMA-IR measure, instead of insulin, produced quite similar results (data not shown). In these analyses, adjustment for leptin also resulted in a notable increase in the main associations, particularly when waist circumference was analyzed.

**Table 3:** Hazards ratios for diabetes according to obesity levels, adjusting for insulin prior to inflammation markers

	**Main exposure**		
	**Overweight **^**a**^	**Obesity **^**b**^	**Waist circumference 4**^**th**^**quartile **^**c**^
	**HR (95%CI)**	**HR (95%CI)**	**HR (95%CI)**
Model 1	2.07 (1.45–2.95)	6.43 (4.48–9.21)	8.30 (5.60–12.28)
Model 1’	1.27 (0.87–1.86	2.67 (1.73–4.13)	3.63 (2.28–5.78)
Model 2	1.23 (0.84–1.81)	2.51 (1.63–3.88)	3.43 (2.16–5.46)
Model 3	1.09 (0.74–1.60)	1.85 (1.20–2.86)	2.54 (1.58–4.08)
Model 4	1.29 (0.86–1.94)	2.48 (1.52–4.05)	4.10 (2.34–7.20)
Model 5	1.17 (0.78–1.76)	2.26 (1.37–3.73)	3.19 (1.58–6.45)

Model 1: Adjusted for age, study center, ethnicity, gender, and family history of diabetes.

Model 1’: Model 1 + ln-insulin.

Model 2: Model 1’ + adiponectin.

Model 3: Model 2 + inflammation score, C3; oxidized LDL, ICAM-1.

Model 4: Model 3 + leptin (gender-specific quartiles).

Model 5: Model 4 + hypertension, non-esterified fatty acids, ln-triglycerides, ln-triglycerides^2^, HDL, BMI, BMI^2^, waist-to-hip ratio, (those not already in the model).

^a^ Hazards ratio of overweight (25 kg/m^2^ ≤ BMI < 30 kg/m^2^) versus BMI < 25 kg/m^2^.

^b^ Hazards ratio of obese (BMI ≥ 30 kg/m^2^) versus BMI < 25 kg/m^2^.

^c^ 4th vs. 1st quartile. In men: waist circumference >102 cm vs. <91 cm; in women: waist circumference >101 cm vs. <84 cm.

No heterogeneity in the pattern of these associations was observed across categories of ethnicity or smoking. A secondary analysis, excluding 27 individuals originally defined as non-cases but with glycemia post 75 g oral glucose load of ≥11.1 mmol/L at ARIC Visit 4, produced similar results (data not shown).

## Discussion

In this middle-age population, obese individuals had a more than 6-fold higher risk of developing diabetes of those with BMI less than 25 kg/m^2^ in models adjusting for age, gender, center, ethnicity, and parental history of diabetes. However, once additional adjustment for adiponectin and inflammation markers was performed, the relative excess risk (1-HR), the measure which best permits comparison of the strength of the main exposure associations following different adjustments, dropped by more than 50%. Similar results were observed when waist circumference and fasting insulin were considered in the models instead of obesity (Table 2). The extent of the reductions seen with adiponectin and inflammation markers was nearly as large as that seen with fasting insulin (Table 3), a surrogate measure of insulin resistance, which is recognized as a major proximal cause of type 2 diabetes.

Obesity has long been considered the major risk factor for the development of diabetes. However, how it exerts its effect has only relatively recently been investigated with robust basic science models, for example, those involving gene knock-out [4]. Once thought to be merely an energy storage site, adipose tissue is now considered the body´s largest endocrine organ, producing a wide variety of intercellular signaling molecules – the so-called adipokines.

Adiponectin is the one of the most expressed of these molecules. Adiponectin has multiple functions, including up-regulation of AMPK, which stimulate glucose uptake into tissue and minimizes liver gluconeogenesis. Additionally, adiponectin has anti-inflammatory and insulin-sensitizing effects [15]. In this community-based sample of U.S. adults [10] and now in many others [16], higher adiponectin levels were associated with a lower incidence of diabetes. With increase in adiposity, adiponectin levels fall. Thus, the marked decrease in risk seen with addition of this adipokine to the model may be explained, at least in part, as mediation of the obesity effect.

Studies investigating various markers of inflammation in different populations have now confirmed our initial demonstration that a low-grade inflammation precedes and predicts diabetes development in middle-age adults [8]. Given the multiple pathways involved in inflammatory processes and the lack of a clear role for specific biomarkers in diabetes etiology, we employed here an inflammation score composed by 6 different markers, as utilized in previous ARIC studies [9]: a mix of a cytokine, four acute-phase reactants, and the leukocyte count. Additionally, we adjusted for other inflammation markers measured more recently in this sample.

How molecular mediators of inflammation might cause diabetes is still open to speculation. We have worked within a conceptual framework based on the necessity, throughout evolution, of growth, development, reproduction on the one hand, and bodily defense for survival, on the other, coupled with the concept that the same internal machinery must adapt to serve these different needs at different times. Thus, metabolism is altered depending on sensed necessities. Within this theory, when the perceived need is growth, development and reproduction, insulin is a key signal. However, when the need is for bodily defense, as signaled by inflammation mediators, metabolism is restructured, and insulin resistance is utilized to minimize energy use in the above-mentioned functions. When inflammation becomes chronic, as seen in obesity, it induces chronic restructuring of metabolic pathways. In fact, multiple effects of inflammation mediators on metabolic parameters which are altered in the progression from obesity to type 2 diabetes have been documented [3,17]. Additionally, adipokine-induced endothelial dysfunction of nutritive vascular beds with subsequent insulin resistance has been postulated [4], perhaps explaining why hypertension and markers of endothelial dysfunction, including those of microvascular circulation [18] have been found to predict the development of diabetes.

Whether the reduction of the association between obesity and type 2 diabetes found with adjustment for inflammation markers is due to mediation or confounding by inflammation cannot be established within our dataset. To a certain extent, it is probably due to both.

In favor of mediation is the fact that obesity is clearly a pro-inflammatory state (obesitis), with changes in adipocyte gene expression and with infiltration of inflammatory cells, most notably macrophages [19] into adipose tissue. Many inflammation markers are produced by activated adipocytes and/or in activated adipose tissue – IL-6, C-reactive protein and complement component C3 being among those that we have analyzed here. Signals generated in adipose tissue, both adipokines and other inflammatory molecules, then lead to striking rises in a series of plasma proteins synthesized by the liver (as fibrinogen and orosomucoid) [9].

However, we [20] and others [21] have shown that low grade chronic inflammation is a strong predictor of weight gain, which argues rather, at least in part, for confounding, in which inflammation would be a common cause of both obesity, and through a separate mechanism, type 2 diabetes. A possible mechanism explaining this confounding is chronic excess, food intake, particularly of pro-inflammatory foodstuffs [22]. Excess food intake goes hand in hand with the development and maintenance of obesity, and excess consumption was shown to be pro-inflammatory [23].

Bench research supports these results. Endoplasmatic reticulum (ER) is a major site for intracellular protein, lipid and sterol synthesis. In balancing intracellular supply and demand in the process of constructing new molecules, the ER has now been shown to be also a major generator of metabolic and inflammatory signals. ER stress is an early consequence of nutrient excess and a cause for the development of inflammation and insulin resistance [24-28]. ER stress, besides inducing the expression of pro-inflammatory cytokines, is also a source of reactive oxygen species, which stimulates pro-inflammatory mediators [24]. Additionally, excess energy supply to mitochondria leads to oxidative stress by uncoupling of the electron chain with release of superoxide [29], a pro-inflammatory event producing an adverse metabolic outcome not only in terms of insulin resistance, but perhaps also in terms of beta cells function [30].

The increased risk for main exposures after addition of leptin to the models merits interpretation. This adipokine has known actions which protect against the development diabetes [31], but its levels are markedly increased in obese subjects, presumably in part to compensate for the state of leptin resistance. In previous analyses, we have shown that leptin is a major risk factor for diabetes in minimally adjusted models, yet appears as a protective factor after adjustment for adiposity, inflammation markers and insulin [11]. Here, we appear to be witnessing a similar phenomenon - the addition of leptin to our adiposity-diabetes analyses reveals a concomitant risk due to leptin resistance. As leptin resistance is strongly correlated with adiposity, the increases seen in the risk measures of adiposity appear to be reflecting this concomitant risk. That the increase in risk seen for adiposity was more pronounced when associations had been previously adjusted for insulin (Table 3) may reflect a greater adjustment for leptin resistance. These variations in relative risk suggest that leptin resistance is an additional important mediator of obesity’s risk, an important question for future epidemiologic investigation.

The role of the additional risk factors – hypertension, non-esterified fatty acids, triglycerides and HDL – in diabetes causation, and thus in confounding or mediating associations seen with the above-mentioned factors, is less clear. Hypertension, as mentioned above, could result, in part, from chronic vasoconstriction caused by adipokine-induced, inflammation-mediated endothelial dysfunction, which in parallel leads to insulin resistance and diabetes. Increased production of angiotensinogen by inflamed adipose tissue is another possible mechanism [32]. Within this context, the decrease in association seen with the addition of hypertension could be seen as marking adipokine mediation of the obesity association. The pattern of high NEFA, high triglyceride with low HDL is characteristic of inflammation, and may be another manifestation of chronic low grade inflammation. Higher NEFA may cause peripheral insulin resistance by interfering with the access of insulin to skeletal muscle or interfering with insulin signaling resulting in reduced glucose transport into muscle; in the liver, high levels lead to excessive endogenous glucose production [33]. In ARIC Study, NEFA was an independent predictor of diabetes [34], and could thus play a mediating role. Our results suggest that processes associated with these factors contribute only minimally, at least for the obesity risk, once insulin has been taken into account.

Our study has some limitations. First, the fact that variables that could be considered potential mediators in this analysis (e.g., adipokines and inflammation markers) were measured at the same moment as the main exposure (obesity) limits our ability to test hypotheses regarding mediation with more sophisticated techniques [35]. Nonetheless, we believe that demonstration of changes in effect measures using a traditional regression approach [36,37] can advance current knowledge at a population level, and may stimulate investigations in datasets with multiple assessments of these factors across time and the employment of more sophisticated modeling techniques. Second, selection bias, either due to participants not returning for follow-up, not having a sample available for measurement, or exclusion of those participants with cardiovascular disease, could conceivably have influenced our results. However, we have little a priori reason to believe that the associations here demonstrated should be stronger or weaker among them. Third, our analyses were based only on fasting samples, and we may not have fully captured the effects of adipokines and inflammation in the postprandial period [23]. If so, our findings might underestimate the size of the reduction in associations reported. The resultant residual confounding might account for some of the remaining risk attributable to obesity in the final model. Fourth, errors in measurements of different analytes may have influenced our results. In this regard, fasting insulin is an imperfect proxy for insulin resistance.

Insulin resistance, along with beta cells failure, is believed to be a major final common pathway from obesity to type 2 diabetes. The reduction in obesity-diabetes relative risk seen with adjustment for insulin is consistent with this view. The fact that markers of chronic inflammation led to a similar relative risk reduction, suggests a major, if not nearly equivalent role for inflammatory pathways in the causation of type 2 diabetes.

Our interpretation of these analyses should not be construed as suggesting a linear pathway (nor multiple linear pathways) from obesity to type 2 diabetes. Undoubtedly the process is a much more complex one, with various processes manifesting along the causal pathways, including, among them, inflammation and insulin resistance/beta cells failure. Given the integration of these processes and the continuum in their installation over time, definition of what comes first is difficult to address epidemiologically and may not be the most relevant question. Population approaches, such as Mendelian randomization, particularly when investigating upstream inflammatory mediators, may complement basic science research in further elucidating the role of chronic inflammation in the progression from obesity to type 2 diabetes [38]. The further elucidation of interactions of inflammatory processes with insulin resistance/beta cells failure, as well as with additional factors of the metabolic syndrome, should advance our understanding of the causes of current epidemic of type 2 diabetes.

## Conclusions

Our findings, when coupled with the literature, support a major role of adiponectin and other inflammatory factors in mediating the progression from obesity to type 2 diabetes.

## Abbreviations

AMK: Activated protein kinase; ANCOVA: Analysis of covariance; ARIC: Atherosclerosis Risk in Communities; C3: Complement C3; ER: Endoplasmatic reticulum; ICAM-1: Intercellular Adhesion Molecule-1; NCEP: Third Report of the National Cholesterol Education Program.

## Competing interests

The authors declare that they have no competing interests.

## Authors’ contributions

All authors contributed significantly to this paper. VCL, MIS and BBD researched data and wrote the manuscript. JSP and JHY researched data, contributed to the discussion and reviewed/edited the manuscript. DC researched data and reviewed/edited the manuscript. CB reviewed/edited the manuscript. All authors read and approved the final manuscript.
